# Reply to Lai and Huang, “Which beta-lactam infusion strategy is superior in ICU pneumonia? A Bayesian network meta-analysis of continuous, extended, and intermittent approaches”

**DOI:** 10.1128/aac.01859-25

**Published:** 2026-03-16

**Authors:** Yixuan Li, Jason A. Roberts, Mohd H. Abdul-Aziz, Fekade B. Sime

**Affiliations:** 1 University of Queensland Centre for Clinical Research, Faculty of Medicine, The University of Queensland 1974, Brisbane, Australia; 2 Herston Infectious Diseases Institute (HeIDI), Metro North Health, Brisbane, Australia; 3 Departments of Pharmacy and Intensive Care Medicine, Royal Brisbane and Women’s Hospital, Brisbane, Australia; 4 Division of Anesthesia Critical Care and Emergency and Pain Medicine, UR UM 103, University of Montpellier, Nimes University Hospital, Nimes, France; 5 School of Pharmacy and Pharmaceutical Sciences, The University of Queensland, Brisbane, Queensland, Australia; Providence Portland Medical Center, Portland, Oregon, USA

## REPLY

We read the comments by Lai and Huang on our study “Continuous or extended versus intermittent infusions of beta-lactam antibiotics in ICU patients with pneumonia: a systematic review and meta-analysis of randomized controlled trials” with interest (1). We appreciate their insightful comments and discussion on key methodological considerations.

Their first methodological consideration relates to the relative advantage of Bayesian versus frequentist statistical frameworks and that of risk difference (RD) versus risk ratio (RR) as effect measures. They suggest that, in the presence of sparse events and zero events in the included trials, a Bayesian framework using RD may be preferable to RR adopted in our original analysis using the frequentist approach. We agree that Bayesian network meta-analysis can provide a valuable complementary perspective, particularly in settings with limited data, and we appreciate the Bayesian reanalysis presented by Lai and Huang. However, our original meta-analysis was designed to align with most previous beta-lactam infusion meta-analyses, for comparison purposes, which usually report relative measures such as RR (2). RR quantifies relative treatment effects, whereas RD reflects absolute changes in risk and is often more robust and clinically interpretable in settings with sparse or zero events (3). However, a key limitation of RD is that it is highly affected by baseline (control) risk, and in settings where baseline risk is highly variable, such as treatment outcome in ICU patients, we would contend that RR is more stable and appropriate to compare the outcome among studies. Importantly, both our original frequentist analysis and the Bayesian RD-based reanalysis by Lai and Huang suggest consistent overall conclusions that the prolonged infusion regimens do not demonstrate statistical superiority over intermittent infusion, or there is insufficient evidence to suggest superiority in terms of mortality or clinical cure in the heterogeneous ICU pneumonia populations.

The second concern relates to the choice between fixed-effects and random-effects models. We agree that random-effects models are generally preferred when substantial between-study heterogeneity is expected. In the present analysis, however, the statistical heterogeneity for the primary outcomes was low to moderate, and meta-analyses applying random-effects models yielded results consistent in both direction and interpretation. Therefore, we used fixed-effects models to achieve greater precision with the limited data from available randomized controlled trials (RCTs), while reporting heterogeneity measures and recognizing residual clinical variability as a study limitation.

The third concern relates to whether extended infusion (EI) and continuous infusion (CI) should be analyzed separately. We agree that EI and CI have different pharmacokinetic profiles that warrant separate analysis. Where data are available, we did include subgroup analyses for EI and CI, albeit the limited data from available RCTs restricted the statistical rigor of analysis, a limitation that also applies equally to the Bayesian analysis of Lai and Huang.

Finally, Lai and Huang note that Bayesian analyses provide clear probability-based interpretations that can complement traditional hypothesis testing. Although their Bayesian network meta-analysis suggests a higher probability of benefit with EI or CI, substantial uncertainty remains due to the limited available data. Their reported credible intervals included zero, suggesting that the potential benefit is not definitive. These findings are consistent with our original conclusion that, while prolonged infusion regimens are pharmacologically attractive, current evidence from randomized controlled trials does not demonstrate a clear clinical benefit for mortality or clinical cure in ICU pneumonia. We agree that the Bayesian analysis represents a meaningful approach; however, its interpretation is constrained by the limited studies and comparability of cohorts between EI and CI, which restricts the reliability of treatment ranking. Accordingly, any inference regarding precise ranking between EI and CI should be interpreted with caution and may be overstated. Nevertheless, the presence of a treatment effect and the associated posterior probabilities of benefit appear reasonable and consistent with the available evidence.

In conclusion, we welcome the Bayesian reanalysis presented by Lai and Huang and believe that it provides a complementary analysis to our original work. Importantly, both analytical approaches result in a similar interpretation: prolonged beta-lactam infusion regimens may offer potential benefit in selected contexts, but the available evidence remains insufficient to demonstrate clear superiority over intermittent infusion for key clinical outcomes. We agree that future meta-analyses would benefit from incorporating Bayesian methods alongside frequentist analyses and, more critically, that adequately powered RCTs are required to reduce uncertainty and inform clinical practice.
